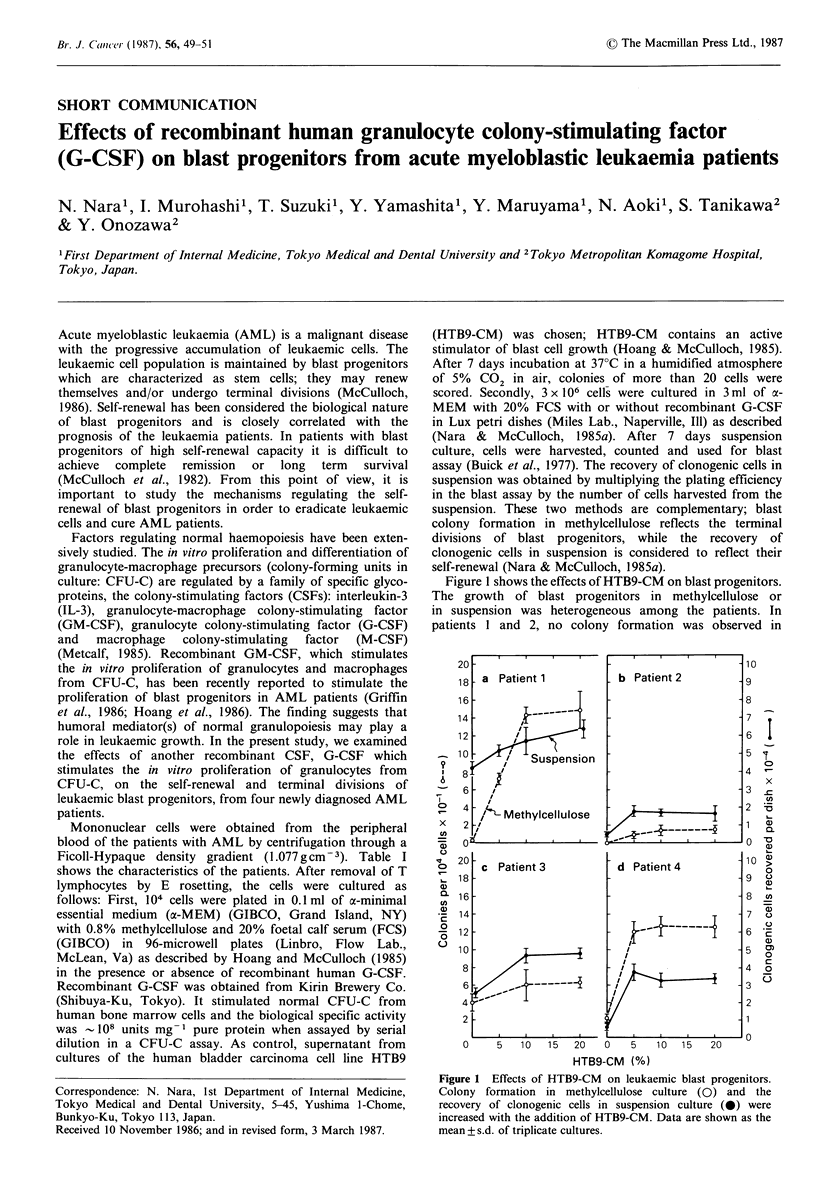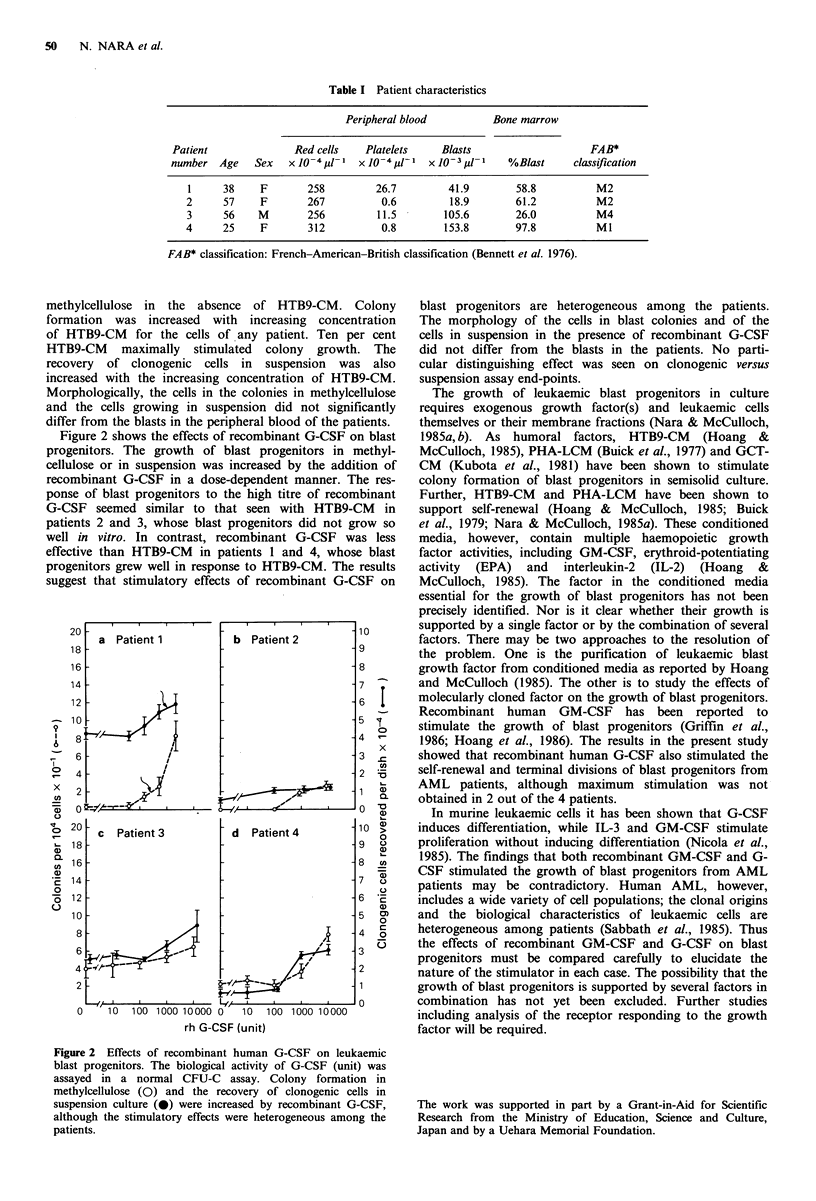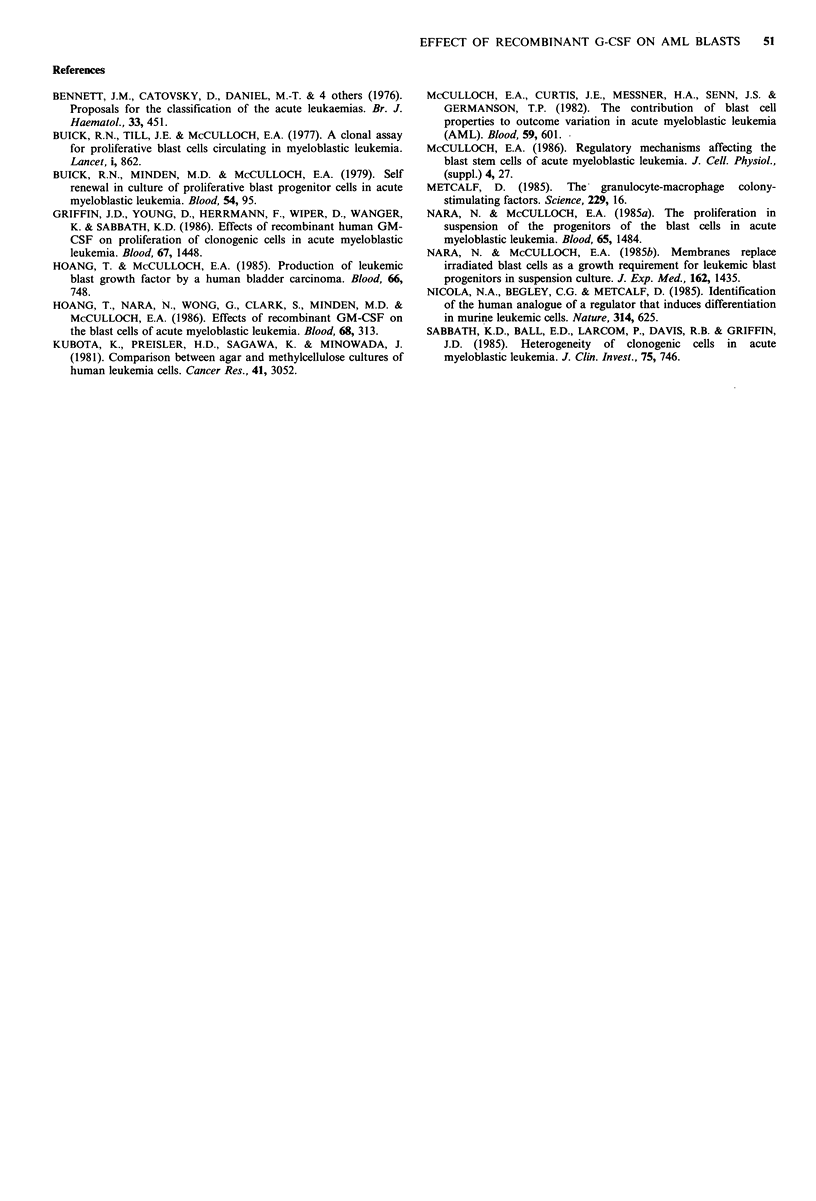# Effects of recombinant human granulocyte colony-stimulating factor (G-CSF) on blast progenitors from acute myeloblastic leukaemia patients.

**DOI:** 10.1038/bjc.1987.151

**Published:** 1987-07

**Authors:** N. Nara, I. Murohashi, T. Suzuki, Y. Yamashita, Y. Maruyama, N. Aoki, S. Tanikawa, Y. Onozawa


Br. J. Cancer (1987), 56, 49–51 © The Macmillan Press Ltd., 1987

SHORT COMMUNICATION

### Effects of recombinant human granulocyte colony-stimulating factor (G-CSF) on blast progenitors from acute myeloblastic leukaemia patients

N. Nara[1], I. Murohashi[1], T. Suzuki[1], Y. Yamashita[1], Y. Maruyama[1], N. Aoki[1], S. Tanikawa[2] & Y. Onozawa[2]

[1]*First Department of Internal Medicine, Tokyo Medical and Dental University and* [2]*Tokyo Metropolitan Komagome Hospital, Tokyo, Japan.*

Acute myeloblastic leukaemia (AML) is a malignant disease with the progressive accumulation of leukaemic cells. The leukaemic cell population is maintained by blast progenitors which are characterized as stem cells; they may renew themselves and/or undergo terminal divisions (McCulloch, 1986). Self-renewal has been considered the biological nature of blast progenitors and is closely correlated with the prognosis of the leukaemia patients. In patients with blast progenitors of high self-renewal capacity it is difficult to achieve complete remission or long term survival (McCulloch *et al.*, 1982). From this point of view, it is important to study the mechanisms regulating the self-renewal of blast progenitors in order to eradicate leukaemic cells and cure AML patients.

Factors regulating normal haemopoiesis have been extensively studied. The *in vitro* proliferation and differentiation of granulocyte-macrophage precursors (colony-forming units in culture: CFU-C) are regulated by a family of specific glycoproteins, the colony-stimulating factors (CSFs): interleukin-3 (IL-3), granulocyte-macrophage colony-stimulating factor (GM-CSF), granulocyte colony-stimulating factor (G-CSF) and macrophage colony-stimulating factor (M-CSF) (Metcalf, 1985). Recombinant GM-CSF, which stimulates the *in vitro* proliferation of granulocytes and macrophages from CFU-C, has been recently reported to stimulate the proliferation of blast progenitors in AML patients (Griffin *et al.*, 1986; Hoang *et al.*, 1986). The finding suggests that humoral mediator(s) of normal granulopoiesis may play a role in leukaemic growth. In the present study, we examined the effects of another recombinant CSF, G-CSF which stimulates the *in vitro* proliferation of granulocytes from CFU-C, on the self-renewal and terminal divisions of leukaemic blast progenitors, from four newly diagnosed AML patients.

Mononuclear cells were obtained from the peripheral blood of the patients with AML by centrifugation through a Ficoll-Hypaque density gradient ($1.077 \mathrm{g cm^{-3}}$). Table I shows the characteristics of the patients. After removal of T lymphocytes by E rosetting, the cells were cultured as follows: First, $10^4$ cells were plated in 0.1 ml of α-minimal essential medium (α-MEM) (GIBCO, Grand Island, NY) with 0.8% methylcellulose and 20% foetal calf serum (FCS) (GIBCO) in 96-microwell plates (Linbro, Flow Lab., McLean, Va) as described by Hoang and McCulloch (1985) in the presence or absence of recombinant human G-CSF. Recombinant G-CSF was obtained from Kirin Brewery Co. (Shibuya-Ku, Tokyo). It stimulated normal CFU-C from human bone marrow cells and the biological specific activity was $\sim 10^8$ units $\mathrm{mg^{-1}}$ pure protein when assayed by serial dilution in a CFU-C assay. As control, supernatant from cultures of the human bladder carcinoma cell line HTB9 (HTB9-CM) was chosen; HTB9-CM contains an active stimulator of blast cell growth (Hoang & McCulloch, 1985). After 7 days incubation at 37°C in a humidified atmosphere of 5% $CO_2$ in air, colonies of more than 20 cells were scored. Secondly, $3 \times 10^6$ cells were cultured in 3 ml of α-MEM with 20% FCS with or without recombinant G-CSF in Lux petri dishes (Miles Lab., Naperville, Ill) as described (Nara & McCulloch, 1985*a*). After 7 days suspension culture, cells were harvested, counted and used for blast assay (Buick *et al.*, 1977). The recovery of clonogenic cells in suspension was obtained by multiplying the plating efficiency in the blast assay by the number of cells harvested from the suspension. These two methods are complementary; blast colony formation in methylcellulose reflects the terminal divisions of blast progenitors, while the recovery of clonogenic cells in suspension is considered to reflect their self-renewal (Nara & McCulloch, 1985*a*).

Figure 1 shows the effects of HTB9-CM on blast progenitors. The growth of blast progenitors in methylcellulose or in suspension was heterogeneous among the patients. In patients 1 and 2, no colony formation was observed in

**Figure 1** Effects of HTB9-CM on leukaemic blast progenitors. Colony formation in methylcellulose culture (○) and the recovery of clonogenic cells in suspension culture (●) were increased with the addition of HTB9-CM. Data are shown as the mean ± s.d. of triplicate cultures.

Correspondence: N. Nara, 1st Department of Internal Medicine, Tokyo Medical and Dental University, 5–45, Yushima 1-Chome, Bunkyo-Ku, Tokyo 113, Japan.
Received 10 November 1986; and in revised form, 3 March 1987.

**Table I** Patient characteristics

| | | | *Peripheral blood* | | | *Bone marrow* | |
|---|---|---|---|---|---|---|---|
| *Patient number* | *Age* | *Sex* | *Red cells* $\times 10^{-4} \mu l^{-1}$ | *Platelets* $\times 10^{-4} \mu l^{-1}$ | *Blasts* $\times 10^{-3} \mu l^{-1}$ | *%Blast* | *FAB* classification* |
| 1 | 38 | F | 258 | 26.7 | 41.9 | 58.8 | M2 |
| 2 | 57 | F | 267 | 0.6 | 18.9 | 61.2 | M2 |
| 3 | 56 | M | 256 | 11.5 | 105.6 | 26.0 | M4 |
| 4 | 25 | F | 312 | 0.8 | 153.8 | 97.8 | M1 |

*FAB** classification: French–American–British classification (Bennett *et al.* 1976).

methylcellulose in the absence of HTB9-CM. Colony formation was increased with increasing concentration of HTB9-CM for the cells of any patient. Ten per cent HTB9-CM maximally stimulated colony growth. The recovery of clonogenic cells in suspension was also increased with the increasing concentration of HTB9-CM. Morphologically, the cells in the colonies in methylcellulose and the cells growing in suspension did not significantly differ from the blasts in the peripheral blood of the patients.

Figure 2 shows the effects of recombinant G-CSF on blast progenitors. The growth of blast progenitors in methylcellulose or in suspension was increased by the addition of recombinant G-CSF in a dose-dependent manner. The response of blast progenitors to the high titre of recombinant G-CSF seemed similar to that seen with HTB9-CM in patients 2 and 3, whose blast progenitors did not grow so well *in vitro*. In contrast, recombinant G-CSF was less effective than HTB9-CM in patients 1 and 4, whose blast progenitors grew well in response to HTB9-CM. The results suggest that stimulatory effects of recombinant G-CSF on

**Figure 2** Effects of recombinant human G-CSF on leukaemic blast progenitors. The biological activity of G-CSF (unit) was assayed in a normal CFU-C assay. Colony formation in methylcellulose (○) and the recovery of clonogenic cells in suspension culture (●) were increased by recombinant G-CSF, although the stimulatory effects were heterogeneous among the patients.

blast progenitors are heterogeneous among the patients. The morphology of the cells in blast colonies and of the cells in suspension in the presence of recombinant G-CSF did not differ from the blasts in the patients. No particular distinguishing effect was seen on clonogenic *versus* suspension assay end-points.

The growth of leukaemic blast progenitors in culture requires exogenous growth factor(s) and leukaemic cells themselves or their membrane fractions (Nara & McCulloch, 1985*a*, *b*). As humoral factors, HTB9-CM (Hoang & McCulloch, 1985), PHA-LCM (Buick *et al.*, 1977) and GCT-CM (Kubota *et al.*, 1981) have been shown to stimulate colony formation of blast progenitors in semisolid culture. Further, HTB9-CM and PHA-LCM have been shown to support self-renewal (Hoang & McCulloch, 1985; Buick *et al.*, 1979; Nara & McCulloch, 1985*a*). These conditioned media, however, contain multiple haemopoietic growth factor activities, including GM-CSF, erythroid-potentiating activity (EPA) and interleukin-2 (IL-2) (Hoang & McCulloch, 1985). The factor in the conditioned media essential for the growth of blast progenitors has not been precisely identified. Nor is it clear whether their growth is supported by a single factor or by the combination of several factors. There may be two approaches to the resolution of the problem. One is the purification of leukaemic blast growth factor from conditioned media as reported by Hoang and McCulloch (1985). The other is to study the effects of molecularly cloned factor on the growth of blast progenitors. Recombinant human GM-CSF has been reported to stimulate the growth of blast progenitors (Griffin *et al.*, 1986; Hoang *et al.*, 1986). The results in the present study showed that recombinant human G-CSF also stimulated the self-renewal and terminal divisions of blast progenitors from AML patients, although maximum stimulation was not obtained in 2 out of the 4 patients.

In murine leukaemic cells it has been shown that G-CSF induces differentiation, while IL-3 and GM-CSF stimulate proliferation without inducing differentiation (Nicola *et al.*, 1985). The findings that both recombinant GM-CSF and G-CSF stimulated the growth of blast progenitors from AML patients may be contradictory. Human AML, however, includes a wide variety of cell populations; the clonal origins and the biological characteristics of leukaemic cells are heterogeneous among patients (Sabbath *et al.*, 1985). Thus the effects of recombinant GM-CSF and G-CSF on blast progenitors must be compared carefully to elucidate the nature of the stimulator in each case. The possibility that the growth of blast progenitors is supported by several factors in combination has not yet been excluded. Further studies including analysis of the receptor responding to the growth factor will be required.

The work was supported in part by a Grant-in-Aid for Scientific Research from the Ministry of Education, Science and Culture, Japan and by a Uehara Memorial Foundation.

### References

BENNETT, J.M., CATOVSKY, D., DANIEL, M.-T. & 4 others (1976). Proposals for the classification of the acute leukaemias. *Br. J. Haematol.*, **33**, 451.

BUICK, R.N., TILL, J.E. & McCULLOCH, E.A. (1977). A clonal assay for proliferative blast cells circulating in myeloblastic leukemia. *Lancet*, **i**, 862.

BUICK, R.N., MINDEN, M.D. & McCULLOCH, E.A. (1979). Self renewal in culture of proliferative blast progenitor cells in acute myeloblastic leukemia. *Blood*, **54**, 95.

GRIFFIN, J.D., YOUNG, D., HERRMANN, F., WIPER, D., WANGER, K. & SABBATH, K.D. (1986). Effects of recombinant human GM-CSF on proliferation of clonogenic cells in acute myeloblastic leukemia. *Blood*, **67**, 1448.

HOANG, T. & McCULLOCH, E.A. (1985). Production of leukemic blast growth factor by a human bladder carcinoma. *Blood*, **66**, 748.

HOANG, T., NARA, N., WONG, G., CLARK, S., MINDEN, M.D. & McCULLOCH, E.A. (1986). Effects of recombinant GM-CSF on the blast cells of acute myeloblastic leukemia. *Blood*, **68**, 313.

KUBOTA, K., PREISLER, H.D., SAGAWA, K. & MINOWADA, J. (1981). Comparison between agar and methylcellulose cultures of human leukemia cells. *Cancer Res.*, **41**, 3052.

McCULLOCH, E.A., CURTIS, J.E., MESSNER, H.A., SENN, J.S. & GERMANSON, T.P. (1982). The contribution of blast cell properties to outcome variation in acute myeloblastic leukemia (AML). *Blood*, **59**, 601.

McCULLOCH, E.A. (1986). Regulatory mechanisms affecting the blast stem cells of acute myeloblastic leukemia. *J. Cell. Physiol.*, (suppl.) **4**, 27.

METCALF, D. (1985). The granulocyte-macrophage colony-stimulating factors. *Science*, **229**, 16.

NARA, N. & McCULLOCH, E.A. (1985*a*). The proliferation in suspension of the progenitors of the blast cells in acute myeloblastic leukemia. *Blood*, **65**, 1484.

NARA, N. & McCULLOCH, E.A. (1985*b*). Membranes replace irradiated blast cells as a growth requirement for leukemic blast progenitors in suspension culture. *J. Exp. Med.*, **162**, 1435.

NICOLA, N.A., BEGLEY, C.G. & METCALF, D. (1985). Identification of the human analogue of a regulator that induces differentiation in murine leukemic cells. *Nature*, **314**, 625.

SABBATH, K.D., BALL, E.D., LARCOM, P., DAVIS, R.B. & GRIFFIN, J.D. (1985). Heterogeneity of clonogenic cells in acute myeloblastic leukemia. *J. Clin. Invest.*, **75**, 746.